# Influenza Vaccine-Averted Illness in Chile, Guyana, and Paraguay During 2013–2018: A Standardized Approach to Assess the Value of Vaccination

**DOI:** 10.1093/infdis/jiaf038

**Published:** 2025-03-10

**Authors:** Jorge H Jara, Sergio Loayza, Francisco Nogareda, Paula Couto, Miguel Angel Descalzo, Anna N Chard, María Fernanda Olivares Barraza, Natalia Vergara Mallegas, Rodrigo A Fasce, Marta Von Horoch, Silvia Battaglia, Elena Penayo, Chavely Montserrat Dominguez, Cynthia Vazquez, Rainier Escalada, Janice Woolford, Fabiana Michel, Rafael Chacon, Ashley Fowlkes, Laura Castro, Martha Velandia-Gonzalez, Marc Rondy, Eduardo Azziz-Baumgartner, Stefano Tempia, Daniel Salas

**Affiliations:** Special Program Comprehensive Immunization, Pan American Health Organization, World Health Organization, Washington, District of Columbia, USA; Special Program Comprehensive Immunization, Pan American Health Organization, World Health Organization, Guatemala City, Guatemala; Special Program Comprehensive Immunization, Pan American Health Organization, World Health Organization, Washington, District of Columbia, USA; Department of Public Health Emergencies, Pan American Health Organization, World Health Organization, Washington, District of Columbia, USA; Department of Public Health Emergencies, Pan American Health Organization, World Health Organization, Washington, District of Columbia, USA; Influenza Division, National Center for Immunization and Respiratory Diseases, US Centers for Disease Control and Prevention, Atlanta, Georgia, USA; Epidemiology Department, Ministry of Health, Santiago, Chile; Epidemiology Department, Ministry of Health, Santiago, Chile; Biomedical Laboratory Department, Viral Diseases Subdepartment, Institute of Public Health, Santiago, Chile; General Directorate of Health Surveillance, Ministry of Public Health and Social Welfare, Asunción, Paraguay; General Directorate of Health Surveillance, Ministry of Public Health and Social Welfare, Asunción, Paraguay; General Directorate of Health Surveillance, Ministry of Public Health and Social Welfare, Asunción, Paraguay; General Directorate of Health Surveillance, Ministry of Public Health and Social Welfare, Asunción, Paraguay; Central Public Health Laboratory, Ministry of Public Health and Social Welfare, Asunción, Paraguay; Communicable Disease Control and Elimination, Pan American Health Organization, World Health Organization, Georgetown, Guyana; Family and Community Health, Pan American Health Organization, World Health Organization, Georgetown, Guyana; Special Program Comprehensive Immunization, Pan American Health Organization, World Health Organization, Santo Domingo, Dominican Republic; Division of Global Health Protection, Centers for Disease Control and Prevention, San Salvador, El Salvador; Influenza Division, National Center for Immunization and Respiratory Diseases, US Centers for Disease Control and Prevention, Atlanta, Georgia, USA; Influenza Division, National Center for Immunization and Respiratory Diseases, US Centers for Disease Control and Prevention, Atlanta, Georgia, USA; Special Program Comprehensive Immunization, Pan American Health Organization, World Health Organization, Washington, District of Columbia, USA; Department of Public Health Emergencies, Pan American Health Organization, World Health Organization, Washington, District of Columbia, USA; Influenza Division, National Center for Immunization and Respiratory Diseases, US Centers for Disease Control and Prevention, Atlanta, Georgia, USA; Global Influenza Program, World Health Organization, Geneva, Switzerland; Special Program Comprehensive Immunization, Pan American Health Organization, World Health Organization, Washington, District of Columbia, USA

**Keywords:** influenza burden, influenza vaccine, influenza epidemiology, averted illness, vaccine effectiveness

## Abstract

**Background:**

To better establish the value of vaccination against influenza viruses, we estimated vaccine-averted influenza illnesses among young children and older adults in Chile, Guyana, and Paraguay.

**Methods:**

We gathered country- and target population-specific data on monthly influenza hospitalizations, vaccine coverage, and vaccine effectiveness from surveillance records and immunization registries during 2013–2018. We applied a static compartmental model to estimate differences in the number influenza-associated respiratory disease events (symptomatic nonhospitalized illnesses, medically attended illnesses, hospitalizations) in the presence and absence of influenza vaccination programs.

**Results:**

Between 2013 and 2018, vaccinating 68% of children aged 6–23 months in Chile averted an annual mean of 14 617 nonhospitalized, 9426 medically attended, and 328 hospitalized influenza illnesses; vaccinating 28% of children aged 6–23 months in Paraguay averted 1115 nonhospitalized, 719 medically attended, and 25 hospitalized influenza illnesses. Vaccinating 59% of older adults in Chile averted an annual mean of 83 429 nonhospitalized, 37 079 medically attended, and 1390 hospitalized influenza illnesses; vaccinating 36% of older adults in Paraguay averted an annual mean of 3932 nonhospitalized, 1748 medically attended, and 66 hospitalized influenza illnesses. In Guyana, a hypothetical campaign vaccinating 30% of children aged <5 years could have prevented an annual 1496 nonhospitalized, 971 medically attended, and 10 hospitalized influenza illnesses. Vaccinating 30% of adults aged ≥65 years could have prevented 568 nonhospitalized, 257 medically attended, and 10 hospitalized influenza illnesses.

**Conclusions:**

Influenza vaccination averted tens of thousands of illnesses and thousands of hospitalizations in Chile and Paraguay; influenza vaccination could have had a proportional benefit in Guyana.

Vaccination is among the most effective strategies for preventing influenza illness and its complications. Influenza vaccines are safe, effective, and routinely used worldwide [1]. While 41 out of 44 (93%) countries in the Americas recommend influenza vaccination, coverage varies throughout the region. Evidence about vaccine-averted illnesses in target populations can inform vaccine policy, communicate benefits of vaccination to the public, and motivate providers to offer more vaccines to persons targeted for vaccination [2].

Influenza infections result in a significant disease burden, affecting individuals of all age groups. Globally, seasonal influenza is estimated to cause 290 000–645 000 respiratory deaths annually [3]. In the Region of the Americas, previous studies have estimated that 716 000–829 000 influenza-associated respiratory hospitalizations and 40 880–160 270 influenza-associated deaths occur annually [4, 5]. The costs associated with influenza illness can be substantial—a recent systematic review of the cost of influenza illness in low- and middle-income countries reported the respective total cost per episode of influenza outpatient visits and hospitalizations was $25.92–$198.13 and $95.15–$2202.74 for children, and $38.17–$164.52 and $282.37–$2729.25 for older adults [6].

To mitigate the morbidity, mortality, and societal cost associated with influenza, the governments of Chile and Paraguay recommend influenza vaccination for all 4 World Health Organization (WHO) target groups—health workers, older adults (the age at which people are considered older varies by country), pregnant women, and persons with comorbidities and underlying conditions. Besides, children aged 6–59 months were included as another important group [7]. During 2013–2018, influenza vaccination coverage among older adults ranged from 52% to 63% in Chile and 33% to 40% in Paraguay while coverage among children ranged from 62% to 74% in Chile and 23% to 32% in Paraguay. Guyana does not currently have a national influenza immunization program with defined target populations and the influenza vaccine is not currently available in the private sector.

Impact assessments compare observed and expected disease burden using data from influenza surveillance and the Essential Program on Immunizations [8]. Through this type of analysis, influenza vaccine coverage and its associated effectiveness can be translated into estimates of vaccine-averted illnesses that better reveal the vaccine's value. These data also serve as the basis to assess the cost-effectiveness and cost-benefit of programs for influenza vaccination target groups.

The Pan American Health Organization (PAHO) Severe Acute Respiratory Infection Network (SARI*net* plus) conducts surveillance for influenza hospitalizations with further monitoring of vaccine effectiveness in a subset of SARI*net* plus hospitals through the Network for the Evaluation of the Effectiveness of the Vaccine in Latin America and the Caribbean for Influenza (REVELAC-i). The present study uses data from these networks to estimate vaccine-averted nonhospitalized influenza illnesses, medically attended illnesses, and hospitalizations by presenting examples of 2 countries with influenza immunization programs (Chile and Paraguay) and 1 without an influenza vaccination program (Guyana).

## METHODS

### Study Population

The study included young children and older adults in Chile and Paraguay during the influenza seasons from 2013 to 2018. In Chile, the target populations for the influenza vaccine include children aged 6–23 months and adults aged 65 years and older. In Paraguay, the target populations for the vaccine include children aged 6–23 months and adults aged 60 years and older. Guyana does not have a national influenza immunization program with defined target populations, nor is the influenza vaccine currently available in the private sector. For our estimation, we included the population estimates for children under 5 years old and adults aged 65 years and older during an average year, 2022.

### Study Design

To estimate the impact of influenza vaccination, we used a static compartmental model adapted from Tokars et al [9], as previously described [10], with parameterized monthly estimates of influenza hospitalizations, vaccination coverage, and effectiveness of influenza vaccine. In countries like Chile and Paraguay, which have influenza immunization programs, the model compares influenza illness estimates under an observed vaccination scenario with modeled estimates of illness that would have occurred in the hypothetical absence of a vaccination campaign. In countries like Guyana, which do not yet have an influenza immunization program, the model compares influenza illness estimates with modeled estimates of illness that would have occurred in the hypothetical presence of a vaccination campaign. Additionally, we used the model to assess how increasing vaccine coverage and utilizing different vaccine deployment strategies would impact averted illness.

### Data Inputs and Sources

#### Target Population

The size of the target population was obtained from the National Institutes of Statistics (Instituto Nacional de Estadística) of Chile and Paraguay [11, 12]. We obtained the target population size for Guyana from the Department of Economic and Social Affairs, United Nations [13].

#### Estimated Monthly Hospitalizations

In Chile and Paraguay, to estimate monthly influenza-associated hospitalizations, we used hospital discharges for which the leading cause of admission was a respiratory system disease diagnosis corresponding to International Classification of Diseases-Tenth Revision (ICD-10) codes J00–J99, by age group. We used the percent positivity for influenza virus obtained from national virologic surveillance by dividing samples positive for any influenza virus by the total number of samples tested. We determined the month- and age-stratified percentage of samples testing positive for the influenza virus by dividing the number of positive samples by the total number of samples analyzed in the same month.

For Guyana, which implemented influenza surveillance in 2023, we used influenza-associated hospitalization estimates previously obtained by Palekar et al [5] available for children younger than 5 years and adults 65 years and older. The total number of influenza hospitalizations were extrapolated from the incidence estimates using the country's total population as of 2022 (808 734 inhabitants, of which 78 980 were children under 5 years and 50 764 were adults aged 65 years and older). In the absence of Guyana's influenza case distribution, which was unavailable for the study period, we obtained the average monthly influenza distribution from 2010 to 2022 using influenza viral circulation distribution from neighboring countries—Suriname and French Guiana—available via FluNet [14].

We used multipliers derived from a Peru study [15] for hospitalized to nonhospitalized influenza illnesses and the proportion of influenza-associated illnesses that were medically attended to transform influenza-associated hospitalizations into nonhospitalized illnesses (defined as mild to moderate symptomatic influenza illness that may be self-limiting or require outpatient/ambulatory medical care, but not hospitalization) and medically attended illnesses (defined as influenza illnesses requiring medical treatment, including hospitalization) for each age group.

#### Vaccine Coverage and Vaccine Effectiveness

The number of people vaccinated against influenza in Chile and Paraguay for each target population per season and month from 2013 to 2018 was collected from administrative influenza vaccination data, electronic records, or coverage surveys. We utilized unpublished data from the REVELAC-i regional influenza vaccine effectiveness estimates, which were calculated using a test-negative design in hospitalized young children (aged 6–24 months) and older adults (aged ≥60 years) in Argentina, Brazil, Chile, Colombia, Paraguay, and Uruguay.

For Guyana, we specified vaccine coverage as 30% of the target population. We obtained vaccine effectiveness from a systematic review and meta-analysis published in 2021 for children under 5 years old [16] and adults aged 65 years and older during the 2017 influenza season [17]. Given year-round circulation of influenza in Guyana, we considered the availability of vaccines for the Northern Hemisphere and the Southern Hemisphere. In the base scenario, the campaign reached 30% coverage over a 4-month campaign (5% coverage in month 1, 10% in month 2, 10% in month 3, 5% in month 4) starting in October (Northern Hemisphere formulation) or in March (Southern Hemisphere formulation).

### Analysis

We used an Excel-based tool developed by PAHO, the World Health Organization, and the US Centers for Disease Control and Prevention (CDC) (PAHO internal document) to run the model, obtain the averted illness estimates, and analyze varying coverage and vaccine deployment scenarios. We calculated confidence intervals using R software through a Monte Carlo simulation of 5000 iterations. If the lower limit of the confidence intervals for vaccine effectiveness was negative, the limit was set to zero [10].

The analytic methods have been previously described [10]. Briefly, in countries with vaccination programs (ie, Chile and Paraguay), the model calculates the monthly incidence of influenza-associated cases by dividing the events observed each month by the population susceptible to that event in the previous month. The monthly susceptible population is estimated as the total population of the target group minus the population that developed influenza and the population effectively vaccinated that month. For the hypothetical scenario without the vaccine, the monthly incidence of influenza-associated cases is calculated, and the susceptible population is estimated as the total population minus only the population that developed influenza, month by month.

For Guyana the number of influenza-associated hospitalizations that could have been prevented through vaccination was estimated as the difference between the observed number of hospitalizations in the absence of vaccination and the expected number of influenza-associated hospitalizations in the presence of vaccination.

We present the results as the number of events averted by vaccination (ie, the number of nonhospitalized influenza illnesses, medically attended influenza illnesses, and influenza-associated hospitalizations) and as the prevented fraction of illnesses, estimated as the percentage of the illnesses averted among illnesses that would have occurred in the absence of vaccination.

#### Additional Vaccine Deployment Scenarios

To show the average annual effect of vaccination campaigns from 2013 to 2018, we calculated the vaccine's effectiveness for that period and monthly averages of hospitalizations and vaccinations. The median population for that period was also taken into consideration.

In addition to the base model, we assessed the effect of increased coverage and different vaccine deployment strategies on the burden of disease averted. The analysis of increased coverage included 40%, 60%, 70%, 75%, 80%, and 90% vaccine coverage. The analysis of campaign deployment scenarios included 3 strategies: in strategy 1, the observed coverage would be achieved in 3 months with the start of the campaign in March (70%, 20%, and 10% of observed coverage in the first, second, and third month, respectively); in strategy 2, the observed coverage would be achieved in 3 months with the start in February (70%, 20%, 10% of observed coverage); and in strategy 3, the observed coverage would be achieved in 2 months with the start in February (80%, 20% of observed coverage).

For Guyana, the baseline scenario used the Northern Hemisphere vaccine and the campaign reached 30% coverage over a 4-month campaign starting in October (5%, 10%, 10%, 5%). The alternative scenarios increased the total coverage to 40%, 50%, 60%, and 70%. We defined 4 alternative deployment strategies. In strategies 1 to 3, the campaign would last 3 months, reaching 50%, 30%, and 20% of the coverage in each consecutive month, but the beginning of the campaign was delayed in the second (November) and third strategies (December). In the fourth strategy, the campaign begins in October, but lasts 10 months, with the same monthly coverage in each month.

### Ethics

We did not collect nominal information or individual identifiers. We obtained the data through routine influenza surveillance, Essential Program on Immunizations, and existing health information systems. The analysis used aggregate data only. The project was submitted to the PAHO Ethical Review Committee and deemed exempt as it did not constitute research with human subjects.

## RESULTS

From 2013 to 2018, Chile had an average annual population of 368 097 children aged 6 to 23 months, with 43 128 influenza cases and 967 hospitalizations. The Southern Hemisphere vaccine used had a median effectiveness of 44% (95% confidence interval [CI], 28–57) and a vaccination coverage of 67.8%. During the same period, 1 956 623 adults aged 65 and older experienced 325 738 influenza cases and 5429 hospitalizations. Their median vaccine effectiveness was 37%, with a coverage of 59.4%. Vaccination campaigns for both age groups began in March (Table 1). Paraguay's median population of children aged 6 to 23 months was 211 329, with 13 730 influenza cases and 308 hospitalizations. Vaccine coverage was 28%. For adults aged 60 and older, the population was 608 850, with 33 985 cases, 566 hospitalizations, and a coverage of 36.4%. Vaccination typically started in April, except in 2016 and 2018, when it began in May (Table 2). Between 2022 and 2023, Guyana had a median population of 78 980 children under 5 years, reporting 10 136 influenza cases and 69 hospitalizations. Among adults aged 65 years and older, with a median population of 50 764, there were 5520 cases and 92 hospitalizations. Hypothetically, using the Northern Hemisphere vaccine, we assumed 30% coverage for both groups, starting in October, with effectiveness at 53% for children and 37% for older adults (Table 3).

**Table 1.** Demographic Background, Viral Circulation, Immunization Campaign, and Influenza Vaccines Used for Children Aged 6–23 Months and Adults 65 Years and Older in Chile, 2013–2018

	2013	2014	2015	2016	2017	2018	Mean 2013–2018
Children aged 6–23 mo							
Population size	377 819	378 069	370 481	372 393	361 748	348 073	368 097
Influenza vaccination coverage, %^a^	74.35	70.87	64.43	67.18	61.75	67.99	67.81
Influenza cases	50 326	22 078	52 041	66 064	27 503	40 755	43 128
Influenza-associated hospitalizations	1129	495	1167	1482	617	914	967
Adults 65 y and older							
Population size	1 774 353	1 837 314	1 906 363	1 985 718	2 070 796	2 165 195	1 956 623
Influenza vaccination coverage, %	58.15	57.38	53.78	52.28	55.36	62.68	59.38
Influenza cases	137 381	138 544	201 626	428 160	596 116	452 597	325 738
Influenza-associated hospitalizations	2290	2309	3360	7136	9935	7543	5429
Viral circulation, %							
Influenza A H1N1pdm09	57.6	1.0	44.6	59.4	0.0	8.1	30.5
Influenza A H3N2	20.5	82.3	31.7	18.4	75.1	73.9	48.0
Influenza B Yamagata	10.8	16.1	13.4	4.3	23.1	16.6	13.6
Influenza B Victoria	11.1	0.7	10.3	18.0	1.8	1.4	7.9
Vaccines							
Formulation	Southern Hemisphere	Southern Hemisphere	Southern Hemisphere	Southern Hemisphere	Southern Hemisphere	Southern Hemisphere	Southern Hemisphere
Component	AH1c, AH3v, BYw	AH1c, AH3t, BYm	AH1c, AH3sw, BYp	AH1c, AH3h, BVb	AH1m, AH3h, BYp	AH1m, AH3si, BYp	…
Month of campaign initiation	March	March	March	March	March	March	…
Regional vaccine effectiveness in children aged 6–23 m, % (95% CI)^b^	35 (3 to 57)	13 (−48 to 49)	45 (26 to 59)	43 (24 to 58)	38 (−8 to 64)	57 (40 to 70)	44 (28 to 57)
Regional vaccine effectiveness in adults aged ≥65 y, % (95% CI)^b^	48 (32 to 60)	50 (32 to 63)	23 (−5 to 43)	42 (23 to 57)	19 (−8 to 39)	47 (41 to 53)	37 (30 to 44)

Abbreviations: AH1c, A/California/7/2009 (H1N1) pdm09-like virus; AH1m, A/MICHIGAN/45/2015 (H1N1) pdm09-like virus; AH3h, A/Hong Kong/4801/2014 (H3N2)-like virus; AH3si, A/Singapore/INFIMH-16-0019/2016 (H3N2)-like virus; AH3sw, A/Switzerland/9715293/2013 (H3N2)-like virus; AH3t, A/Texas/50/2012 (H3N2)-like virus; AH3v, A/Victoria/361/2011 (H3N2)-like virus; BYm, B/Massachusetts/2/2012-like virus; BYp, B/Phuket/3073/2013-like virus; BYw, B/Wisconsin/1/2010-like virus; BVb, B/Brisbane/60/2008-like virus; CI, confidence interval.

^a^Children who have received 1 dose of the influenza vaccine are considered completely vaccinated if they have been previously vaccinated and 2 doses if it is the first time they are vaccinated.

^b^Regional effectiveness obtained through REVELAC-i. Source: Ministry of Health of Chile.

**Table 2.** Demographic Background, Viral Circulation, Immunization Campaign, and Influenza Vaccines Used for Children Aged 6–23 Months and Adults 60 Years and Older in Paraguay, 2013–2018

	2013	2014	2015	2016	2017	2018	Mean, 2013–2018
Children aged 6–23 m							
Population size	210 437	210 809	211 173	211 555	211 860	212 139	211 329
Influenza vaccination coverage, %^a^	28.9	31.6	31.7	23.1	28.6	24.1	28.0
Influenza cases	9588	8594	16 543	17 394	10 705	19 558	13 730
Influenza-associated hospitalizations	215	193	371	390	240	439	308
Adults 60 y and older							
Population size	551 527	573 367	596 043	619 530	643 829	668 805	608 850
Influenza vaccination coverage, %	39.7	38.7	33.2	37.9	35.6	34.0	36.4
Influenza cases	25 107	14 432	41 351	38 381	41 705	42 936	33 985
Influenza-associated hospitalizations	418	241	689	640	695	716	566
Viral circulation, %							
Influenza A H1N1pdm09	8.5	4.6	32.3	63.8	0.0	8.3	21.8
Influenza A H3N2	63.8	69.5	47.8	0.3	72.0	60.7	50.3
Influenza B	27.7	25.9	19.8	31.6	28.0	31.0	28.0
Vaccines							
Formulation	Southern Hemisphere	Southern Hemisphere	Southern Hemisphere	Southern Hemisphere	Southern Hemisphere	Southern Hemisphere	Southern Hemisphere
Component	AH1c, AH3v, BYw	AH1c, AH3t, BYm	AH1c, AH3sw, BYp	AH1c, AH3h, BVb	AH1m, AH3h, BYp	AH1m, AH3si, BYp	…
Month of campaign initiation	April	April	April	May	April	May	…
Regional vaccine effectiveness in children aged 6–23 m, % (95% CI)^b^	35 (3 to 57)	13 (−48 to 49)	45 (26 to 59)	43 (24 to 58)	38 (−8 to 64)	57 (40 to 70)	44 (28 to 57)
Regional vaccine effectiveness in adults aged ≥60 y, % (95% CI)^b^	48 (32 to 60)	50 (32 to 63)	23 (−5 to 43)	42 (23 to 57)	19 (−8 to 39)	47 (41 to 53)	37 (30 to 44)

Abbreviations: AH1c, A/California/7/2009 (H1N1)pdm09-like virus; AH1m, A/MICHIGAN/45/2015 (H1N1) pdm09-like virus; AH3h, A/Hong Kong/4801/2014 (H3N2)-like virus; AH3SI, A/Singapore/INFIMH-16-0019/2016 (H3N2)-like virus; AH3sw, A/Switzerland/9715293/2013 (H3N2)-like virus; AH3t, A/Texas/50/2012 (H3N2)-like virus; AH3v, A/Victoria/361/2011 (H3N2)-like virus; BYm, B/Massachusetts/2/2012-like virus; BYp, B/Phuket/3073/2013-like virus; BYw, B/Wisconsin/1/2010-like virus; BVb, B/Brisbane/60/2008-like virus; CI, confidence interval.

^a^Children who have received 1 dose of the influenza vaccine are considered completely vaccinated if they have been vaccinated previously and 2 doses if it is the first time they are vaccinated.

^b^Regional effectiveness obtained through REVELAC-i. Source: Ministry of Health of Paraguay.

**Table 3.** Demographic Background, Viral Circulation, Immunization Campaign, and Influenza Vaccines Used for Children Aged < 5 Years and Adults 65 Years and Older in Guyana, 2022–2023

	2022–2023
Children aged < 5 y	
Population size	78 980
Influenza vaccination coverage, %^a^	30.0
Influenza cases	10 136
Influenza-associated hospitalizations	69
Adults 65 y and older	
Population size	50 764
Influenza vaccination coverage, %	30.0
Influenza cases	5520
Influenza-associated hospitalizations	92
Viral circulation, %	
Influenza A H1N1pdm09	27.5
Influenza A H3N2	47.6
Influenza B	24.2
Vaccines	
Formulation	Northern Hemisphere
Component	AH1v, AH3d, BVa
Month of campaign initiation	October
Vaccine effectiveness in children aged 6–23 mo, % (95% CI) [16]	53 (47 to 59)
Vaccine effectiveness in adults aged ≥60 y, % (95% CI) [17]	37 (30 to 44)

Abbreviations: AH3d, A/Darwin/9/2021 (H3N2)-like virus; AH1v, A/Victoria/2570/2019 (H1N1)pdm09-like virus; BVa, B/Austria/1359417/2021 (B/Victoria lineage)-like virus;

CI, confidence interval.

^a^Hypothetical influenza coverage using Northern Hemisphere vaccine composition.

### Vaccine-Averted Influenza Illness Among Children Aged 6–23 Months in Chile and Paraguay During 2013 and 2018

In Chile, influenza vaccination averted a mean of 14 617 nonhospitalized influenza illnesses, 9426 medically attended illnesses, and 328 hospitalizations per year among the 368 097 children aged 6–23 months (Table 4). In Paraguay, influenza vaccination averted a mean of 1115 nonhospitalized influenza illnesses, 719 medically attended illnesses, and 25 hospitalizations per year among the 211 329 children aged 6–23 months. The averted influenza illnesses varied substantially by year; the greatest prevented fraction was estimated in 2018 (Chile 35.6%; 95% CI, 27.3%–42.1% and Paraguay 11.8%; 95% CI, 9.0%–13.9%), and the lowest prevented fraction was observed in 2014 (Chile 7.2%; 95% CI, 0.0%–23.4% and Paraguay 2.7%; 95% CI, 0.0%–8.9%).

**Table 4.** Number of Influenza-Associated Events Averted and Prevented Fraction by the Influenza Vaccine in Children Aged 6–23 Months in Chile and Paraguay in 2013–2018

	Averted Number (95% CI)			Prevented Fraction, % (95% CI)
	Nonhospitalized Illnesses	Medically Attended Illnesses	Hospitalizations	
Chile				
2013	12 997 (3814–23 211)	8381 (2419–14 955)	292 (86–518)	20.8 (7.1–31.5)
2014	1550 (0–6229)	1000 (0–3995)	35 (0–138)	7.2 (.0–23.4)
2015	18 517 (11 571–25 942)	11 941 (7432–16 662)	415 (260–571)	26.2 (18.4–32.8)
2016	21 835 (12 860–31 573)	14 081 (8239–20 315)	490 (291–696)	24.8 (16.5–31.8)
2017	6231 (1071–11 472)	4018 (697–7364)	140 (24–252)	18.5 (3.8–28.8)
2018	21 997 (14 804–29 924)	14 185 (9490–19 220)	493 (334–655)	35.6 (27.3–42.1)
Total	93 770 (55 576–210 928)	61 838 (36 409–138 844)	2103 (1249–4751)	26.9 (18.5–46.0)
Annual mean	14 617 (9540–20 153)	9426 (6155–12 943)	328 (217–442)	25.6 (18.5–31.6)
Paraguay				
2013	720 (229–1169)	464 (147–753)	16 (5–26)	8.0 (2.8–12.1)
2014	222 (0–795)	143 (0–513)	5 (0–18)	2.7 (.0–8.9)
2015	1270 (845–1700)	819 (543–1099)	28 (19–38)	7.3 (5.1–9.2)
2016	926 (587–1261)	597 (378–814)	21 (13–28)	5.4 (3.6–7.0)
2017	620 (0–1127)	400 (0–725)	14 (0–25)	5.8 (.0–9.8)
2018	2435 (1744–3102)	1570 (1124–1997)	55 (40–68)	11.8 (9.0–13.9)
Total	7719 (5167–10 840)	5091 (3405–7158)	173 (115–245)	9.0 (6.2–12.3)
Annual mean	1115 (761–1479)	719 (490–956)	25 (17–33)	8.0 (5.8–9.9)

### Burden of Influenza Disease Averted in Older Adults in Chile and Paraguay

In Chile, influenza vaccination averted a mean of 83 429 nonhospitalized influenza illnesses, 37 079 medically attended illnesses, and 1390 hospitalizations per year among 1 956 623 people 65 years and older (Table 5). In Paraguay, influenza vaccination averted a mean of 3932 nonhospitalized illnesses, 1748 medically attended illnesses, and 66 hospitalizations per year among 608 850 people 60 years and older. The averted influenza illnesses varied substantially by year; the greatest prevented fraction was observed in 2014 in Chile (27.9%; 95% CI, 20.5%–33.6%) and in 2013 in Paraguay (17.5%; 95% CI, 13.2%–21.0%) and the lowest prevented fraction was observed in 2017 in Chile (9.8%; 95% CI, 0.0%–17.8%) and in 2015 in Paraguay (5.7%; 95% CI, 0.8%–9.6%).

**Table 5.** Number of Influenza-Associated Events Averted and Prevented Fraction by the Influenza Vaccine in Adults Aged 65 Years and Older in Chile and Adults Aged 60 Years and Older in Paraguay in 2013–2018

	Averted Number (95% CI)			Prevented Fraction, % (95% CI)
	Nonhospitalized Illnesses	Medically Attended Illnesses	Hospitalizations	
Chile				
2013	47 711 (30 683–69 854)	21 205 (13 727–31 089)	795 (547–1024)	26.4 (19.8–31.6)
2014	52 740 (33 058–77 625)	23 440 (14 698–34 732)	879 (582–1150)	27.9 (20.5–33.6)
2015	29 536 (6857–54 990)	13 127 (3017–24 716)	492 (115–846)	13.2 (3.4–20.7)
2016	113 199 (63 281–175 016)	50 311 (28 268–78 109)	1887 (1103–2605)	21.7 (14.0–27.6)
2017	62 559 (0–132 808)	27 804 (0–59 923)	1043 (0–2067)	9.8 (.0–17.8)
2018	74 173 (0–158 334)	32 966 (0–71 008)	1236 (0–2476)	14.7 (.0–25.6)
Total	574 183 (445 119–741 419)	259 186 (201 066–334 669)	9570 (7404–12 351)	23.4 (19.2–28.3)
Annual mean	83 429 (60 177–116 616)	37 079 (26 921–51 898)	1390 (1113–1657)	21.0 (17.6–24.0)
Paraguay				
2013	3955 (2588–5772)	1758 (1150–2564)	66 (46–84)	17.5 (13.2–21.0)
2014	2824 (1815–4163)	1255 (806–1849)	47 (32–61)	16.7 (12.2–20.1)
2015	2338 (290–4412)	1039 (129–1975)	39 (5–68)	5.7 (.8–9.6)
2016	4891 (2846–7405)	2174 (1258–3302)	82 (49–110)	12.6 (8.0–16.1)
2017	2585 (0–5311)	1149 (0–2368)	43 (0–82)	5.9 (.0–10.7)
2018	6807 (5098–9280)	3025 (2261–4133)	113 (97–130)	15.2 (13.6–16.7)
Total	25 253 (20 297–31 123)	11 399 (9170–14 050)	421 (337–520)	11.6 (9.6–13.9)
Annual mean	3932 (2855–5478)	1748 (1268–2440)	66 (53–78)	11.3 (9.4–12.9)

### Simulation of Influenza Averted Burden in Guyana

In 2022, the total population in Guyana was 808 734, of whom 78 980 were children younger than 5 years and 50 764 were adults aged 65 years and older (Supplementary Table 3). Averted influenza illnesses from a hypothetical vaccination campaign in Guyana are shown in Table 6. Vaccination in advance of the Northern Hemisphere influenza season (ie, October–January) would have averted more influenza illnesses than vaccinating in advance of the Southern Hemisphere season (ie, March–June); in children younger than 5 years, vaccination before the Northern Hemisphere season would have prevented an estimated 1496 nonhospitalized illnesses, 971 medically attended illnesses, and 10 hospitalizations, with a prevented fraction of 14.8% (Table 6). In older adults (aged ≥65 years), vaccination before the Northern Hemisphere season would have prevented 568 nonhospitalized illnesses, 257 medically attended illnesses, and 10 hospitalizations, with a prevented fraction of 10.3%.

**Table 6.** Comparison of Prevented Fraction and Averted Cases by Influenza Vaccination With Vaccines of Northern and Southern Hemisphere Composition During an Average Year in Guyana

	Averted Cases (95% CI)				Prevented Fraction, % (95% CI)
	Nonhospitalized Illnesses	Medically Attended Illnesses	Hospitalizations	Overall	
Children younger than 5 y					
Northern Hemisphere vaccine	1496 (803.0–3981.8)	971 (521.8–2589.8)	10 (7.6–13.0)	1507 (812.2–3991.0)	14.8 (13.2–16.2)
Southern Hemisphere vaccine	1161 (605.2–3290.5)	754 (390.6–2162.9)	8 (5.7–10.5)	1169 (610.9–3300.3)	11.5 (9.7–13.4)
People 65 y and older					
Northern Hemisphere vaccine	568 (350.3–1068.2)	257 (157.9–482.9)	10 (7.1–12.0)	578 (358.7–1078.1)	10.3 (8.6–11.9)
Southern Hemisphere vaccine	439.0 (13.3–40.2)	198 (119.0–382.2)	7 (5.4–9.5)	446 (269.4–855.3)	8.0 (6.5–9.5)

### Additional Vaccine Scenarios

**Figure 1.**
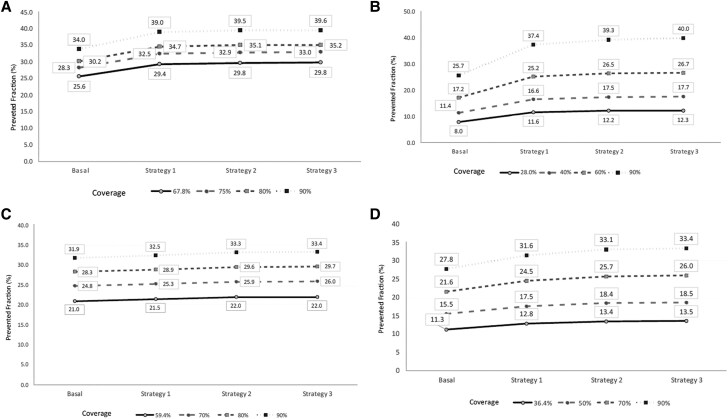
Basal coverage metrics are as follows: (*A*) Actual coverage achieved over 9 months (March-December). (*B*) Coverage achieved over 10 months (April-December). (*C*) Coverage achieved over 5 months (March-July). (*D*) Coverage achieved over 9 months (April-December). In Strategy 1, the observed coverage would be achieved over 3 months with the following distribution: 70% coverage in the first month, 20% in the second month, and 10% in the third month. In Strategy 2, the observed coverage would also be achieved in 3 months, following the same distribution of 70%, 20%, and 10%, but with the campaign's start date brought forward by 1 month. In Strategy 3, the observed coverage was achieved in 2 months with the distribution of 80% coverage in the first month and 20% in the second month, also with the start of the campaign moved forward by 1 month.

The number of averted illnesses increased under all modeled scenarios of increased vaccination coverage in Chile and Paraguay, both for children and older adults (Supplementary Table 1 and Figure 1). In Chile, all 3 modeled vaccine deployment strategies with earlier start dates and more concentrated campaigns increased the number of vaccine-averted nonhospitalized illnesses, medically attended illnesses, and hospitalizations, although strategies 2 (advancing the start of the campaign by 1 month and concentrating deployment to 3 months) and 3 (advancing the start of the campaign by 1 month and concentrating deployment to 2 months) outperformed the baseline strategy by a few thousand averted illnesses (ie, percentage change from baseline: +16.1% for strategy 2 and +16.4% for strategy 3 among children; +4.4% for strategy 2 and +4.7% for strategy 3 among older adults). Similarly, in Paraguay, all 3 modeled strategies also increased averted illnesses among children and adults; strategy 3 resulted in the most averted nonhospitalized illnesses (27 032), medically attended illnesses (12 014), and hospitalizations (451) in older adults (Supplementary Table 1).

In an average influenza season in Guyana, strategy 1 (concentrating deployment to 3 months) would achieve the maximum potential of the campaign, but the impact of the baseline strategy (deployment across 4 months) and strategy 2 (concentrating deployment to 3 months and delaying the campaign 1 month) would be minimally different. Strategy 1 and a 70% coverage of the influenza vaccination campaign could avoid 25 hospitalizations and 3610 nonhospitalized illnesses in children younger than 5 years and 23 hospitalizations and 1371 nonhospitalized illnesses in adults older than 65 (Supplementary Table 2).

## DISCUSSION

We estimated that influenza vaccination campaigns in Chile averted more than 25% of the mean annual disease burden in young children and more than 20% among older adults. In Paraguay, influenza vaccination reduced nearly 10% of the influenza burden among young children and over 10% among older adults. For Guyana, a hypothetical influenza vaccination campaign with 30% coverage could have prevented nearly 15% of the disease burden in children and 10% in adults aged 65 and older in an average year.

Differences in averted influenza illnesses observed by year in Chile and Paraguay were due largely to annual variations in influenza vaccine effectiveness, coverage achieved, timeliness of the start of the campaign, and speed at which final coverage was achieved. In Chile, we found that increasing vaccine coverage had the greatest impact on averted illness; by comparison, achieving coverage in the first 3 months of the campaign and starting the campaign earlier would not have substantially impacted the reduction in disease burden. In Paraguay, vaccine coverage and campaign timing would substantially impact the vaccine-averted disease burden among older adults. An earlier start to the campaign to achieve coverage prior to peak influenza circulation would have prevented an additional 2% of the disease burden. Such an impact was not observed among children, as the complete vaccination scheme in a large proportion of children requires 2 influenza vaccine doses. In both countries, the vaccine-averted disease burden is concentrated in the months with the highest health care demand during the influenza season, reducing the upsurge in health systems.

In countries like Guyana, where influenza surveillance was recently implemented and the influenza vaccine has not yet been introduced, modeling the disease burden and the potential impact of a vaccination campaign might aid policymakers and the public to understand the value of influenza vaccination. Our analysis suggests that introducing the influenza vaccine could reduce the disease burden, especially in children under 5 years. Vaccinating in advance of the Northern Hemisphere season would likely have a greater impact than vaccinating in advance of the Southern Hemisphere season, considering the timing of the influenza season in Guyana. Early vaccination with high, concentrated coverage is more effective than vaccinating later in the year, although improving influenza surveillance is recommended to verify these results. With a population of 800 000, Guyana has a low burden of influenza disease, resulting in a relatively low vaccine impact in absolute numbers; however, because the influenza surveillance system is in its early stages, the burden may increase with more years of data, which will lead to an increase in the vaccine impact. Our analysis highlights the benefits of seasonal vaccination in preventing severe cases in Guyana, supporting the introduction of the influenza vaccine in high-risk groups. In addition to children and older adults, the WHO also recommends influenza vaccination for health professionals, pregnant women, and people with comorbidities; exploring the value of vaccinating these other vaccine target groups in Guyana may be considered as well [7]. Other factors to consider prior to influenza vaccine introduction include cost of a government-subsidized vaccination program, cost-effectiveness of vaccination, acceptability of the vaccine by the population, and capacity of the health system to deliver the vaccines [18].

The results of influenza vaccine impact analyses have several potential uses, one of them being to facilitate the delivery of information to the public regarding the value of vaccinating communities, information that may be easier for the public to understand than vaccine effectiveness. For example, Paraguay used the results of this analysis for a communication strategy aimed at increasing influenza vaccination coverage by raising awareness among the target population (https://www.facebook.com/share/r/Dk5UCvMzZH1u7vke/?mibextid=0VwfS7). Moreover, the burden of influenza-associated disease prevented by vaccination is necessary for economic and cost-effectiveness analyses of vaccines.

This analysis has several limitations. First, we estimated burden of influenza disease based on respiratory disease symptoms and not the wider spectrum of illnesses and complications attributed to influenza. This may result in undercounting more than half of the total influenza disease burden in some groups, such as children. Therefore, estimates of vaccine-averted influenza illnesses are likely underestimated. Second, these results are based on regional vaccine effectiveness data obtained through the REVELAC-i network, which includes Chile and Paraguay, but may still vary locally depending on the circulation of different influenza virus types and subtypes. Third, effectiveness estimates show the effectiveness in preventing hospitalizations (SARI) but not outpatient cases or cases in the community. Therefore, averted respiratory influenza illnesses could be overestimated. Fourth, vaccine effectiveness was assumed to remain constant throughout the season, with protection lasting until the beginning of the next season, regardless of when it was administered. Fifth, the potential indirect effects of the vaccination program or herd immunity were not considered, as integrating these factors into existing evidence at the regional level has many challenges [19]. Sixth, to estimate the burden of the milder disease, we used multipliers based on a cohort in Peru. Due to potential societal and cultural differences in health care access, estimates may not accurately reflect Chile, Guyana, and Paraguay [20]. Finally, to model the potential averted burden scenarios in Guyana, model inputs were based on secondary sources of information and assumptions that may not accurately represent the reality in the country. For example, we assumed that influenza incidence and epidemic curves in Guyana were similar to those in Suriname and French Guyana, neighboring Caribbean countries, and assumed the vaccine effectiveness was the same for all seasons and all disease severity levels. Additionally, the population size for averted illness among children includes all children aged <5 years, including children 0–6 months who are too young for influenza vaccination; therefore, the estimate of averted illness among the target population (children 6–59 months) is an overestimate.

## CONCLUSION

Influenza vaccination campaigns were estimated to reduce influenza-associated disease burden in Chile and Paraguay during 2013–2018 by 10%–25%, and influenza vaccination could have similar impacts if introduced in Guyana. In addition to increasing vaccine coverage, the timing of influenza vaccine campaigns is crucial to their impact, with the greatest benefits observed when campaigns start as soon as the vaccine is available and high coverage is achieved within 2 to 3 months of campaign start. Estimates of vaccine-averted illnesses can be used to inform the public about the value of influenza vaccination for the community, to motivate providers to offer more influenza vaccination to those eligible, as inputs for economic and cost-effectiveness analyses of vaccines, and to help policy makers determine the optimal timing and deployment of vaccine campaigns. In conclusion, while influenza vaccination campaigns effectively reduce the burden of influenza-associated disease, ongoing surveillance and long-term evaluations are crucial for accurately assessing their effectiveness and impact.

## Supplementary Data

Supplementary materials are available at *The Journal of Infectious Diseases* online (http://jid.oxfordjournals.org/). Supplementary materials consist of data provided by the author that are published to benefit the reader. The posted materials are not copyedited. The contents of all supplementary data are the sole responsibility of the authors. Questions or messages regarding errors should be addressed to the author.

## Supplementary Material

jiaf038_Supplementary_Data

## Notes

***Acknowledgments.*** We thank Olaya Astudillo for the review, editing, and input to this article. The authors acknowledge the contributions of SARI*net* working groups, REVELAC-i, the Pan American Health Organization country focal points, and Chile, Paraguay, and Guyana Ministries of Health staff.

***Author contributions.*** J. H. J. had full access to all the data in the study and is responsible for its integrity and the accuracy of the data analysis. J. H. J., S. L., F. N., P. C., and M. A. D. contributed conceptualization, methodology, and supervision. J. H. J., S. L., M. O., N. V., R. A. F., M. V. H., S. B., E. P., C. V., J. W., M. A. D., and P. C. performed data curation. S. L. and J. H. J. contributed software and performed formal analysis. J. H. J., S. L., and F. N. performed project administration. J. H. J., S. L., M. O., N. V., R. A. F., M. V. H., S. B., E. P., C. M. D., C. V., J. W., M. A. D., and P. C. performed validation. J. H. J., S. L., F. N., and P. C. contributed visualization. J. H. J., F. N., and P. C. wrote the original draft. All authors contributed to review and editing, and approved the final version of the manuscript.

***Financial support*.** No financial support was received for this work.

***Supplement sponsorship.*** This article appears as part of the supplement “Advancing Respiratory Virus Surveillance and Preparedness in the Americas: A Decade of SARInet's Impact,” sponsored by the Pan American Health Organization.

***Potential conflicts of interest*.** All authors: No reported conflicts of interest. All authors have submitted the ICMJE Form for Disclosure of Potential Conflicts of Interest. Conflicts that the editors consider relevant to the content of the manuscript have been disclosed.

## References

[1] World Health Organization . Seasonal influenza vaccination: developing and strengthening national programmes—policy brief, 2023. https://www.who.int/publications/i/item/9789240084636. Accessed 28 August 2024.

[2] Pan American Health Organization . Immunization. https://www.paho.org/en/topics/immunization#:∼:text=The%20Special%20Program%20Comprehensive%20Immunization,elimination%20strategies%20to%20improve%20the. Accessed 28 August 2024.

[3] Iuliano AD, Roguski KM, Chang HH, et al Estimates of global seasonal influenza-associated respiratory mortality: a modelling study. Lancet 2018; 391:1285–300. 29248255 10.1016/S0140-6736(17)33293-2 PMC5935243

[4] Cheng PY, Palekar R, Azziz-Baumgartner E, et al Burden of influenza-associated deaths in the Americas, 2002–2008. Influenza Other Respir Viruses 2015; 9(Suppl 1):13–21. 26256291 10.1111/irv.12317 PMC4549098

[5] Palekar RS, Rolfes MA, Arriola CS, et al Burden of influenza-associated respiratory hospitalizations in the Americas, 2010–2015. PLoS One 2019; 14:e0221479. 31490961 10.1371/journal.pone.0221479 PMC6730873

[6] Gharpure R, Chard AN, Cabrera Escobar M, et al Costs and cost-effectiveness of influenza illness and vaccination in low- and middle-income countries: a systematic review from 2012 to 2022. PLoS Med 2024; 21:e1004333. 38181066 10.1371/journal.pmed.1004333 PMC10802964

[7] World Health Organization . Vaccines against influenza: WHO position paper–May 2022. Wkly Epidemiol Rec 2022; 97:185–208.

[8] World Health Organization . Essential programme on immunization. https://www.who.int/teams/immunization-vaccines-and-biologicals/essential-programme-on-immunization. Accessed 28 August 2024.

[9] Tokars JI, Rolfes MA, Foppa IM, Reed C. An evaluation and update of methods for estimating the number of influenza cases averted by vaccination in the United States. Vaccine 2018; 36:7331–7. 30327213 10.1016/j.vaccine.2018.10.026 PMC6666399

[10] Chard AN, Machingaidze C, Loayza S, et al Estimating averted illnesses from influenza vaccination for children and pregnant women—El Salvador, Panama, and Peru, 2011–2018. Vaccine 2024; 42:125861. 38584055 10.1016/j.vaccine.2024.04.007 PMC11455982

[11] Instituto Nacional de Estadística Chile . Proyecciones de población. https://www.ine.gob.cl/estadisticas/sociales/demografia-y-vitales/proyecciones-de-poblacion. Accessed 22 August 2023.

[12] Instituto Nacional de Estadística Paraguay . Resultados finales del censo nacional de población y viviendas 2022. CNVP 2022. https://www.ine.gov.py/publication-single.php?codec=257. Accessed 4 September 2023.

[13] United Nations, Department of Economic and Social Affairs, Population Division . World population prospects 2024, 2024. https://population.un.org/wpp/. Accessed 24 April 2024.

[14] World Health Organization . Global influenza program. FluNet functions. https://www.who.int/tools/flunet. Accessed 24 October 2024.

[15] Tinoco YO, Azziz-Baumgartner E, Uyeki TM, et al Burden of influenza in 4 ecologically distinct regions of Peru: household active surveillance of a community cohort, 2009–2015. Clin Infect Dis 2017; 65:1532–41. 29020267 10.1093/cid/cix565 PMC5850002

[16] Boddington NL, Pearson I, Whitaker H, Mangtani P, Pebody RG. Effectiveness of influenza vaccination in preventing hospitalization due to influenza in children: a systematic review and meta-analysis. Clin Infect Dis 2021; 73:1722–32. 33772586 10.1093/cid/ciab270

[17] Rondy M, El Omeiri N, Thompson MG, et al Effectiveness of influenza vaccines in preventing severe influenza illness among adults: a systematic review and meta-analysis of test-negative design case-control studies. J Infect 2017; 75:381–94. 28935236 10.1016/j.jinf.2017.09.010 PMC5912669

[18] Guillaume D, Meyer D, Waheed DN, et al Factors influencing the prioritization of vaccines by policymakers in low- and middle-income countries: a scoping review. Health Policy Plan 2023; 38:363–76. 36315461 10.1093/heapol/czac092

[19] Lipsitch M . Challenges of vaccine effectiveness and waning studies. Clin Infect Dis 2019; 68:1631–3. 30204853 10.1093/cid/ciy773 PMC6495011

[20] Tempia S, Walaza S, Moyes J, et al Quantifying how different clinical presentations, levels of severity, and healthcare attendance shape the burden of influenza-associated illness: a modeling study from South Africa. Clin Infect Dis 2019; 69:1036–48. 30508065 10.1093/cid/ciy1017 PMC7804385

