# Supplementary material for: Influenza Vaccine-Averted Illness in Chile, Guyana, and Paraguay During 2013–2018: A Standardized Approach to Assess the Value of Vaccination
Source: J Infect Dis. 2025 Mar 10;231(Suppl 2):S133–43. doi: 10.1093/infdis/jiaf038 (PMC11891998; doi:10.1093/infdis/jiaf038)
Supplement: jiaf038_Supplementary_Data [file jiaf038_supplementary_data.docx]

**Table S1: Influenza-associated events averted in different scenarios of influenza vaccine campaign coverage or deployment strategies for children aged 6–23 months in Chile and Paraguay and older adults in Chile and Paraguay, the average of years 2013–2018**

|  | **Non-hospitalized illnesses averted** | **Medically attended illnesses averted** | **Hospitalizations averted** | **Percentage change from baseline** |
| --- | --- | --- | --- | --- |
| **Chile** |  |  |  |  |
| **Children aged 6–23 months** |  |  |  |  |
| *Baseline (67.8%)* | *14,617* | *9426* | *328* | *--* |
| 75% coverage | 16,166 | 10,425 | 363 | + 10.6% |
| 80% coverage | 17,244 | 11,120 | 387 | + 17.9% |
| 90% coverage | 19,399 | 12,510 | 435 | + 32.6% |
| Strategy 1 | 16,798 | 10,832 | 377 | + 14.9% |
| Strategy 2 | 17,005 | 10,966 | 381 | + 16.1% |
| Strategy 3 | 17,046 | 10,992 | 382 | + 16.4% |
| **Adults aged ≥65 years** |  |  |  |  |
| *Baseline (59.4%)* | *83,429* | *37,079* | *1390* | *--* |
| 70% coverage | 98,346 | 43,709 | 1639 | + 17.9% |
| 80% coverage | 112,396 | 49,954 | 1873 | + 34.7% |
| 90% coverage | 126,445 | 56,198 | 2107 | + 51.6 |
| Strategy 1 | 85,092 | 37,819 | 1418 | + 2.0% |
| Strategy 2 | 87,059 | 38,693 | 1451 | + 4.4% |
| Strategy 3 | 87,367 | 38,830 | 1456 | + 4.7% |
| **Paraguay** |  |  |  |  |
| **Children aged 6–23 months** |  |  |  |  |
| *Baseline (27.9%)* | *6192* | *3993* | *139* | *--* |
| 40% coverage | 9401 | 6062 | 211 | + 51.8% |
| 60% coverage | 14,102 | 9093 | 316 | + 127.3% |
| 90% coverage | 21,152 | 13,640 | 474 | + 241.0% |
| Strategy 1 | 8668 | 5589 | 194 | + 39.6% |
| Strategy 2 | 9375 | 6046 | 210 | + 51.1% |
| Strategy 3 | 9423 | 6077 | 211 | + 51.8% |
| **Adults aged ≥60 years** |  |  |  |  |
| *Baseline (36.4%)* | *23,401* | *10,400* | *390* | *--* |
| 40% coverage | 32,240 | 14,329 | 537 | + 37.7% |
| 60% coverage | 45,136 | 20,061 | 752 | + 92.8% |
| 90% coverage | 58,032 | 25,792 | 967 | + 147.9% |
| Strategy 1 | 24,782 | 11,014 | 413 | + 5.9% |
| Strategy 2 | 26,607 | 11,825 | 443 | + 13.6% |
| Strategy 3 | 27,032 | 12,014 | 451 | + 15.6% |

Analyses based on regional vaccine effectiveness data for 2013–2018. In strategy 1, the observed coverage would be achieved in 3 months (70% coverage the first month, 20% the second month, 10% the third month); in strategy 2, the observed coverage would be achieved in 3 months (70%, 20%, 10% coverage) with the start of the campaign brought forward 1 month from the baseline. In strategy 3, the observed coverage was achieved in 2 months (80%, 20% coverage), with the campaign starting 1 month from the baseline.

**Table S2: Prevented fraction and averted non-hospitalized and hospitalized influenza infections with different influenza vaccine coverage and deployment strategies in children younger than 5 years and adults aged 65 years and older in Guyana during an average year**

|  | **Children (aged <5 years)** | | | | | **Adult (aged ≥65 years)** | | | | |
| --- | --- | --- | --- | --- | --- | --- | --- | --- | --- | --- |
|  | **Coverage** | | | | | **Coverage** | | | | |
|  | **30%**  **(baseline)** | **40%** | **50%** | **60%** | **70%** | **30%**  **(baseline)** | **40%** | **50%** | **60%** | **70%** |
| **Prevented fraction** | | | | | | | | | | |
| **Baseline strategy** | **14.8%** | **19.7%** | **24.6%** | **29.5%** | **34.4%** | **10.3%** | **13.7%** | **17.2%** | **20.6%** | **24.0%** |
| Strategy 1 | 15.3% | 20.4% | 25.4% | 30.5% | 35.6% | 10.6% | 14.2% | 17.7% | 21.3% | 24.8% |
| Strategy 2 | 14.7% | 19.6% | 24.5% | 29.4% | 34.2% | 10.2% | 13.6% | 17.1% | 20.5% | 23.9% |
| Strategy 3 | 13.5% | 18.0% | 22.5% | 27.0% | 31.5% | 9.4% | 12.5% | 15.7% | 18.8% | 22.0% |
| Strategy 4 | 9.7% | 12.9% | 16.2% | 20.2% | 22.6% | 6.7% | 9.0% | 11.2% | 14.2% | 15.7% |
| **Averted hospitalized infections** | | | | | | | | | | |
| **Baseline strategy** | **10** | **14** | **17** | **20** | **24** | **9** | **13** | **16** | **19** | **22** |
| Strategy 1 | 11 | 14 | 18 | 21 | 25 | 10 | 13 | 16 | 20 | 23 |
| Strategy 2 | 10 | 14 | 17 | 20 | 24 | 9 | 13 | 16 | 19 | 22 |
| Strategy 3 | 9 | 12 | 16 | 19 | 22 | 9 | 12 | 14 | 17 | 20 |
| Strategy 4 | 7 | 9 | 11 | 14 | 16 | 6 | 8 | 10 | 13 | 14 |
| **Averted non-hospitalized infections** | | | | | | | | | | |
| **Baseline strategy** | **1496** | **1995** | **2494** | **2993** | **3491** | **568** | **757** | **947** | **1136** | **1325** |
| Strategy 1 | 1547 | 2063 | 2579 | 3095 | 3610 | 588 | 784 | 980 | 1176 | 1371 |
| Strategy 2 | 1488 | 1984 | 2480 | 2975 | 3471 | 565 | 753 | 941 | 1129 | 1318 |
| Strategy 3 | 1370 | 1827 | 2283 | 2740 | 3197 | 519 | 692 | 866 | 1039 | 1212 |
| Strategy 4 | 983 | 1311 | 1638 | 2050 | 2294 | 372 | 496 | 620 | 784 | 867 |

In the baseline scenario, the campaign achieved 30% coverage over a 4-month period (5% coverage in the first month, 10% in the second and third months, and 5% in the fourth month). In strategy 1, a 3-month campaign (50%, 30%, 20% of the coverage each month), starting the month the vaccine is available; In strategy 2: a 3-month campaign (50%, 30%, 20% of the coverage each month), starting 1 month after the vaccine is available; In strategy 3: a 3-month campaign (50%, 30%, 20% of the coverage each month), starting 2-month after the vaccine is available; and in strategy 4: a 10-month campaign (10% of the coverage each month), starting since the vaccine is available.

**Table S3: Estimation of influenza burden in Guyana during 2022**

|  | **Total population** | **Age <5 years** | **Age 5–64 years** | **Age ≥65 years** |
| --- | --- | --- | --- | --- |
| **Population (2022)** | 808,734 | 78,980 | 678,990 | 50,764 |
| **Incidence  (per 100,000 inhabitant)** | 37 | 87 | 20 | 182 |
| **Number of hospitalizations (95% CI)** | 297 (150–812) | 69 (46–104) | 136 (87–208) | 92 (17–500) |
